# TLC-Embedded PDMS-Based Wearable Skin Patch for Vein Visualization Applications

**DOI:** 10.3390/polym18161934

**Published:** 2026-08-07

**Authors:** Mayesha Binte Mahmud, Yalda Chehrehsaz, Rafaela Aguiar, Anthony V. Tuccitto, Lei Zhang, Vivian Luu, Isabella Terefenko, Katherine Jia, Xia Wei Shen, Christopher T. Chan, Patrick C. Lee

**Affiliations:** 1 Multifunctional Composites Manufacturing Laboratory, Department of Mechanical and Industrial Engineering, University of Toronto, 5 King’s College Road, Toronto, ON M5S 3G8, Canada; 2 Division of Nephrology, Department of Medicine, University Health Network, 200 Elizabeth Street, 8N Room 846, Toronto, ON M5G 2C4, Canada

**Keywords:** thermochromic liquid crystals, wearable skin patch, vein visualization, temperature-responsive materials, PDMS elastomer, arteriovenous fistula monitoring

## Abstract

A flexible skin patch based on thermochromic liquid crystals (TLCs) embedded within a polydimethylsiloxane (PDMS) elastomer is presented for passive visualization of skin–vein thermal contrasts. Five ternary TLC formulations composed of cholesteryl oleyl carbonate (COC), cholesteryl nonanoate (CN), and cholesteryl benzoate (CB) were systematically engineered to tune mesophase stability and helical pitch sensitivity within the physiological temperature range (32–37 °C). Small compositional adjustments (≤1 wt.%) produced pronounced and predictable shifts in color-play bandwidth and optical sensitivity, governed by thermally induced contraction of the cholesteric helix. Patterned TLC microdomains were integrated onto optically absorptive PDMS substrates and encapsulated within a transparent PDMS overlayer, yielding thin, mechanically compliant, and breathable films with uniform thermal transmission. Optical spectroscopy, optical microscopy, and RGB analysis revealed linear wavelength–temperature relationships (R^2^ > 0.9) and red-to-green sensitivities of up to ~87 nm °C^−1^, demonstrating high thermochromic sensitivity within the physiological skin temperature range. Mechanical characterization confirmed skin-matched elasticity, while preliminary sterilization studies indicated that the films retained their mechanical properties following ethanol immersion and UV exposure. As a feasibility study, the results demonstrate the potential of a compositionally tunable thermochromic elastomer platform for visualizing physiological skin temperature variations associated with superficial veins, providing a basis for further development toward wearable vein visualization for self-cannulation in home hemodialysis and related thermal sensing applications.

## 1. Introduction

Home hemodialysis provides significant clinical and quality-of-life benefits for patients with end-stage renal disease [1]. Compared with conventional in-centre hemodialysis, home-based dialysis allows longer and more frequent treatments that more closely replicate continuous renal filtration, leading to improved blood pressure control, enhanced solute clearance, reduced cardiovascular burden, and improved survival outcomes [2,3,4,5,6,7]. These benefits are particularly important given that cardiovascular mortality among hemodialysis patients remains 10–20 times higher than in the general population [8,9,10]. Home hemodialysis also reduces travel burden, improves scheduling flexibility and sleep quality, and minimizes exposure to infectious diseases [11,12]. Despite these advantages, adoption remains limited, largely due to challenges associated with vascular access cannulation. Long-term hemodialysis typically relies on arteriovenous fistulas (AVF) or grafts that require precise, repetitive needle insertion, making self-cannulation technically demanding and anxiety-provoking [4,13,14,15,16]. Improper cannulation increases the risk of infiltration, hematoma, thrombosis, and infection, while existing vein-localization approaches—including palpation, ultrasound guidance, or permanent skin markings—are constrained by variability in reliability, cost, accessibility, or patient acceptability [15,17]. Ultrasound systems, although effective, are impractical for routine home use, whereas tattoos or surgical markers are permanent and aesthetically undesirable [18,19]. Consequently, there is a clear need for a low-cost, non-invasive, and user-friendly vein-visualization tool to improve cannulation accuracy in home settings. Table 1 summarizes the strengths and limitations of current approaches for AVF visualization in the context of home hemodialysis. Although several imaging modalities can identify vascular structures, they generally require external hardware, trained operators, or indirect image interpretation. These limitations motivate the development of a passive, wearable sensor without external electronics.

One promising strategy for developing wearable vein-visualization sensors builds on the influence of superficial blood flow on local skin temperature. Warm blood (~37 °C) flowing through veins near the epidermis elevates the temperature of the overlying skin, producing measurable thermal contrasts that depend on vessel diameter, depth, and flow rate [31,32,33]. Draper et al. demonstrated that veins located ~2 mm beneath the skin surface generate distinct thermal signatures detectable at the skin interface [33]. Numerical and experimental studies by Ratovoson et al. further showed that vessels within 1–2 mm of the surface induce faster and greater skin heating than surrounding tissue, effectively revealing underlying vasculature through thermal gradients [31]. Consistently, Gao et al. quantified skin temperature variations associated with vessel proximity, reporting contrasts in the range of ~0.4–1.2 °C between regions directly above major vessels and adjacent tissue [32]. Together, these findings confirm that subcutaneous blood flow produces predictable thermal gradients suitable for non-invasive vein visualization.

Thermotropic cholesteric liquid crystals provide an effective platform for translating subtle temperature variations into visible optical signals. They are typically composed of elongated, rod-like mesogenic molecules that form a highly ordered crystalline lattice at low temperatures. Upon heating above the melting point, this rigid order gradually breaks down, and the material passes through intermediate liquid-crystalline phases, often forming a smectic phase first. With further temperature increase, the material transitions from the smectic to the cholesteric (chiral nematic) phase. Smectic liquid crystals are relatively solid-like, with molecules aligned and organized in layers, whereas cholesteric liquid crystals are more fluid, retaining local alignment while adopting a continuous helical twist [34,35,36]. The distance over which the molecular director completes a full 360° rotation is called the helical pitch, which determines the wavelength of light selectively reflected by the cholesteric phase. In thermochromic liquid crystals (TLCs), the helical pitch is highly temperature-sensitive, producing reversible color changes within a narrow mesophase window; beyond this range, the structure becomes isotropic and the color disappears [37,38]. These thermally driven optical transitions provide the physical basis for engineering TLC formulations that respond selectively within narrow temperature windows.

Building on these fundamental thermochromic principles, cholesteric liquid crystals offer a uniquely tunable platform for wearable thermal sensing through compositional control of their helical pitch and mesophase stability [38,39,40]. In practice, individual cholesteryl esters are combined to precisely position the active color-play window within a narrow target temperature range. Among commonly used constituents, cholesteryl oleyl carbonate (COC), cholesteryl nonanoate (CN), and cholesteryl benzoate (CB) share a common mesogenic core but differ in chain flexibility and aromaticity, enabling fine modulation of pitch sensitivity and thermal response through small compositional adjustments [40,41]. Because the selective reflection efficiency of TLCs is inherently limited, integration onto optically absorptive substrates is essential to maximize visible contrast [32,40,42].

Polydimethylsiloxane (PDMS) provides a soft, biocompatible, and skin-conformal substrate that ensures efficient thermal coupling for wearable sensing [43,44,45,46,47]. Its flexibility, ease of fabrication, and compatibility with microstructuring enable reliable integration of functional elements [48,49,50]. Recent studies have demonstrated the integration of TLCs with PDMS and other elastomeric substrates for wearable temperature mapping and physiological monitoring applications [32,40]. However, these systems were primarily developed for spatial temperature measurement and thermal monitoring rather than direct visualization of vascular structures. Embedded TLCs within PDMS can transduce subtle skin-surface temperature variations into spatially resolved color patterns, making this approach particularly effective for vein visualization, where small physiological temperature differences must be detected. In this work, we develop a flexible, PDMS-based thermochromic skin patch for passive visualization of superficial veins using naturally occurring skin–vein thermal contrasts. Unlike previously reported TLC/PDMS platforms designed primarily for epidermal temperature mapping [40] or high-resolution thermochromic thermal imaging [32], the proposed system is specifically engineered to enhance vascular thermal signatures and provide direct visual localization of superficial veins on the skin surface. Compositionally tuned ternary cholesteric liquid crystal mixtures are integrated into mechanically compliant PDMS films, demonstrating a scalable wearable platform for vein visualization.

## 2. Experimental Section

### 2.1. Materials

PDMS (Sylgard™ 184, Dow Inc., Midland, MI, USA) was used as the primary substrate and encapsulating layer material due to its biocompatibility, flexibility, and tunable mechanical properties. To enhance the visual contrast of the thermochromic color transition, optically absorptive black pigments were incorporated into the PDMS substrate layer to create a sufficiently dark background for the TLC layer. Carbon black (CBk-lampblack pigment; Inoxia Ltd., Ashford, Kent, UK) and iron oxide (IO-Iron Oxide Pigment Black 11; The Earth Pigments Company, LLC, Easton, PA, USA) were selected as candidate additives because both are widely used black pigments capable of imparting a dark appearance to polymer matrices, including PDMS. TLC crystal components, COC, CN, and CB, were purchased from Sigma-Aldrich (Oakville, ON, Canada) and used as received. Xylene (Sigma-Aldrich, Oakville, ON, Canada) was used as a solvent for dissolving and blending the TLC formulations. Commercial silica gel desiccant was obtained from Wisesorbent Technology LLC (Marlton, NJ, USA) and used for moisture control during material storage and processing.

### 2.2. Fabrication Method

**Figure 1:**
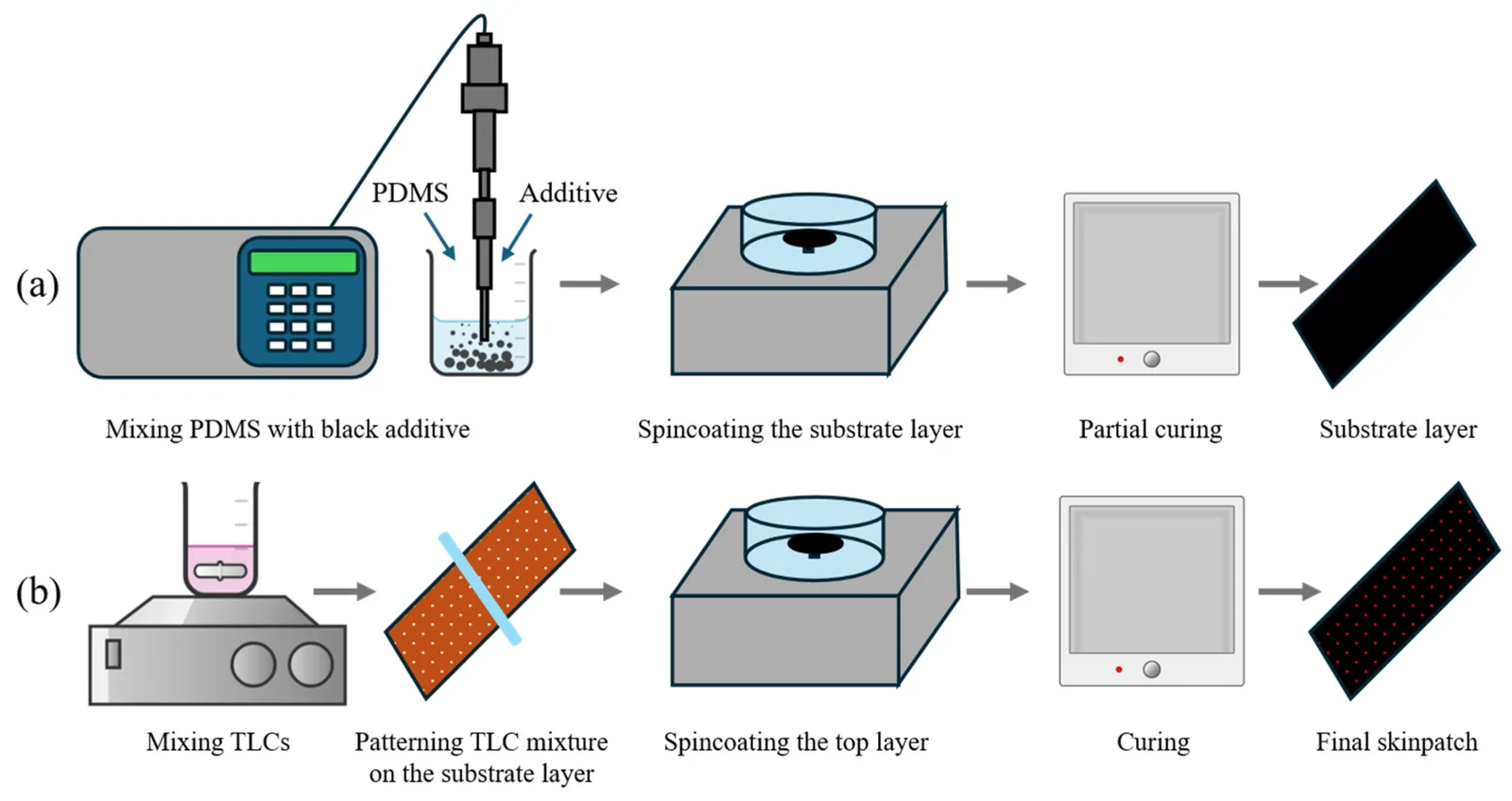
Schematic representation of (**a**) the fabrication process of the black substrate layer, and (**b**) the subsequent integration of the TLC layer to obtain the final patch.

The skin-patch sensor was fabricated using a staged, precision-controlled workflow designed to ensure uniformity, repeatability, and functional integration. Fabrication began with hand-mixing (using glass rod) 30 mL of PDMS base with optically absorptive pigments (1 wt.% carbon black (CBk) or 4 wt.% iron oxide (IO)) in a 50 mL glass beaker to form a homogeneous pre-blend. The selected pigment loadings were adopted from previously reported studies to achieve an effective dark background for enhanced optical contrast [32,40]. This was followed by ultrasonic dispersion to achieve complete particle distribution. To control thermal loading during sonication, the mixture was placed in a water bath, with the probe positioned approximately one-third into the liquid column. Sonication was performed at 85% amplitude in pulsed mode (10 s on/10 s off) for six cycles of 2 min each to break up agglomerates without overheating. After dispersion, the curing agent was added at a 40:1 base-to-cross-linker ratio and mixed for 10 min to ensure uniform cross-linker distribution. The formulation then underwent two brief high-intensity sonication steps (100% amplitude, continuous, 1 min each) to finalize mixing. The resulting mixture was degassed under vacuum for 15 min prior to film formation. Substrate fabrication was subsequently performed via a two-step spin-coating process on 25 × 75 mm glass slides. The first PDMS layer was spin-coated at 1000 rpm for 2.5 min and cured at 95 °C for 18 min. A second layer was applied under identical spin conditions and cured for an extended period (35 min) to produce a uniform, partially cured elastomeric substrate. Partial curing was employed to promote effective transfer of the TLC layer and strong interfacial bonding with the subsequent encapsulation layer. TLC mixtures (total mass: 1 g per formulation) were prepared by weighing COC, CN, and CB according to the desired compositional ratios. The solid components were heated to 60 °C, after which 0.1 mL of xylene was added to promote dissolution and homogenization. The mixture was manually stirred with a glass rod for 10 min to ensure complete blending and compositional uniformity. The resulting TLC formulations were patterned onto the partially cured PDMS substrate using a laser-cut stencil with 500 µm diameter apertures arranged at a uniform center-to-center spacing of 500 µm. Optical and microscopic images of the stencil are provided in the Supplementary Information (Figure S1). The TLC material was deposited through the stencil using a blade-coating technique and briefly cooled in a refrigerator at 4 °C for 5 min to stabilize the patterned dots prior to stencil removal. The patterned TLC layer was then encapsulated with a protective PDMS overcoat (10:1 base-to-cross-linker ratio), spin-coated at 1000 rpm for 5 min and cured at 100 °C for 40 min. This fabrication process yielded a thin, flexible, and mechanically robust skin-mounted temperature-sensing patch (Figure 1). Figure S2 shows the schematic of the three-layer architecture, consisting of a black substrate, thermochromic liquid crystal layer, and transparent protective layer, along with the final assembled wearable thermochromic skin patch. The final fabricated skin patch, including the patterned TLC layer and protective top layer, exhibited an overall thickness of 229.26 ± 6.9 μm, and an overall lateral dimension of 75 × 25 mm.

### 2.3. Mechanism of Thermochromism in TLC Mixtures

**Figure 2:**
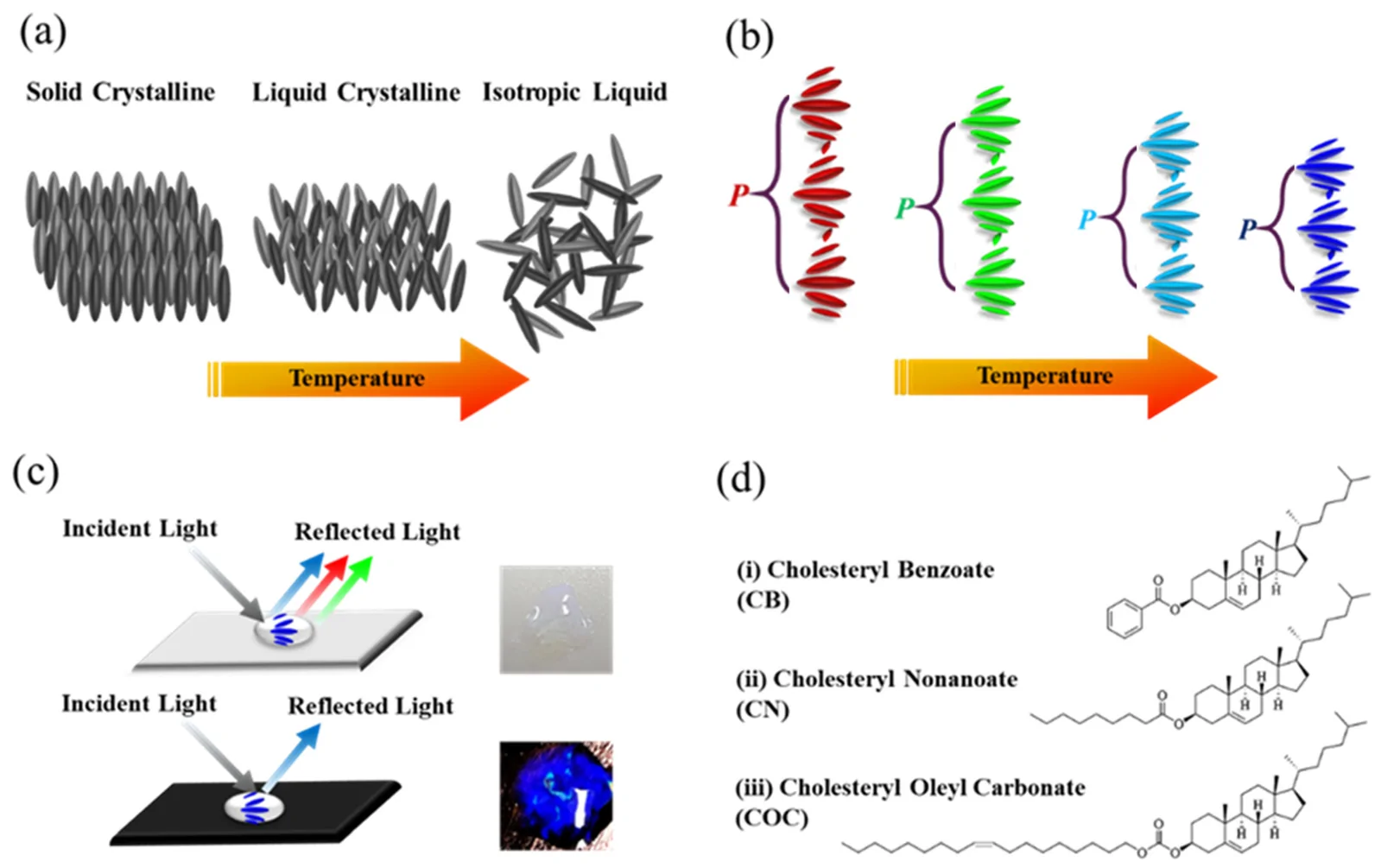
Schematic illustrations of (**a**) phase changes in liquid crystalline materials through temperature increase, (**b**) mechanism of thermochromic liquid crystals through pitch alterations with increasing temperature, and corresponding reflected wavelengths within visible range, (**c**) black substrate facilitates enhanced color visualization of TLCs, and (**d**) chemical structures of the cholesteryl ester derivatives employed in this research. (i) Cholesteryl Benzoate (C_34_H_50_O_2_), (ii) Cholesteryl Nonanoate (C_36_H_62_O_2_), and (iii) Cholesteryl Oleyl Carbonate (C_46_H_80_O_3_).

Figure 2 summarizes the fundamental mechanisms that govern the thermochromic response of the cholesteric liquid crystal mixtures used in this study. As illustrated in Figure 2a, at low temperatures, TLC materials exist in a crystalline solid phase, where molecules exhibit both positional and orientational order. In this state, molecular mobility is insufficient to support the formation of a long-range helical superstructure, and therefore selective reflection and thermochromism are absent [36]. Upon heating above the melting temperature, the system enters the liquid crystalline phase where it transforms from highly ordered smectic phase to cholesteric (chiral nematic) mesophase. Within the cholesteric phase, the molecules adopt a helical arrangement whose pitch determines the selective reflection of wavelength [34,51]. As shown in Figure 2b, heating induces a progressive contraction of the helical pitch (*P*), shifting the reflected wavelength (*λ*) from red to green to blue in accordance with Bragg’s law, *λ* = *nP*, where *n* is the refractive index. This temperature-dependent pitch modulation underpins the reversible color transitions leveraged for thermal sensing. Figure 2c highlights the role of substrate optics: a black background efficiently absorbs transmitted light, thereby enhancing the visibility and saturation of the reflected structural color—a critical factor for maximizing contrast during vein visualization on human skin. Finally, Figure 2d presents the chemical structures of the three cholesteryl esters—COC, CN, and CB that were selected for their complementary phase-transition temperatures and molecular packing characteristics. Their synergistic combination enables precise tuning of the cholesteric pitch within the narrow physiological temperature window relevant to superficial vein detection.

### 2.4. Characterization

The DSC measurements were conducted following heating–cooling-heating protocols using TA DSC 250 (TA Instruments-Waters, LLC, New Castle, DE, USA): the sample was heated to 70 °C at a rate of 30 °C min^−1^, held isothermally at 70 °C for 5 min, cooled to −10 °C at 30 °C min^−1^, and held isothermally for 10 min. This was followed by two additional heating–cooling cycles between −10 and 70 °C at a rate of 20 °C min^−1^. The transformation temperatures are calculated from the 2nd heating cycle. The DSC measurements were performed on at least three identical samples for each formulation, and the mesophase transformation temperatures are reported as mean ± standard deviation (SD). The corresponding mesophase transformation temperatures and SD values are provided in Table S1 of the Supplementary Information.

The wavelength corresponding to the maximum reflected intensity (peak reflected wavelength) was measured using a Nix Spectro 2 spectrophotometer (Nix Sensor Ltd., Hamilton, ON, Canada) over the visible range of 400–700 nm. Measurements were performed on a temperature-controlled heating stage integrated with an optical microscope, within a temperature range of 32–41 °C.

Optical microscopy (OM) was employed to determine the thermochromic temperature range of the TLC mixtures using an Olympus BX51P microscope (Olympus, Tokyo, Japan) equipped with a Linkam Scientific Instruments DSC stage (Linkam Scientific Instruments Ltd., Redhill, UK). Precise control of the heating and cooling rates was achieved using liquid nitrogen. The samples were prepared by depositing the TLC mixtures onto DSC pans coated with black ink to enhance contrast and facilitate visualization of color changes. The samples were first heated from room temperature to 50 °C at a rate of 50 °C min^−1^ and held isothermally for 10 min to ensure thermal equilibration. Subsequently, the samples were cooled to 25 °C at a controlled rate of 0.2 °C min^−1^ using liquid nitrogen, during which the thermochromic color transitions were recorded. Both images and videos of the color evolution were captured during the cooling process. The OM measurements were performed on at least three identical samples for each formulation. The measured color-transition temperatures exhibited good reproducibility, with variations ranging from 0.1 to 0.3 °C among the replicate samples. One representative OM video from each formulation was selected for the RGB analysis.

Moisture permeability of the sensor patches was evaluated by monitoring water vapor transmission through the films using a desiccant-based mass uptake approach [52]. Briefly, 1 g of silica gel desiccant was placed inside glass vials, which were then sealed by covering the vial opening with the sensor patch. The seal was reinforced using paraffin film wrapped around the vial neck to eliminate edge leakage. The mass of each vial was measured at approximately 24 h intervals over a period of five days under ambient laboratory conditions (temperature ~ 23 °C, relative humidity ~ 50%). Desiccant mass gain was determined from the increase in vial mass over time, calculated relative to the initial mass of the vial containing dried desiccant. The WVTR was calculated from the slope of the linear regression of desiccant mass gain over time, according to the exposed film area, and reported as g m^−2^ day^−1^.

Tensile testing was performed using an Instron 6800 universal testing machine (Instron, Norwood, MA, USA) in accordance with ASTM D882 standards for thin films. Rectangular specimens measuring 75 mm × 25 mm, with a gauge length of 50 mm, were tested using a 5 kN load cell at a constant crosshead speed of 1 mm min^−1^.

Thermal imaging was performed using an HT-18 Thermal Imaging system (Dongguan Xintai Instrument and Meter Co., Ltd., Dongguan, China) with a resolution of 22 × 160 pixels to evaluate the surface temperature distribution of the samples. The films were placed on a temperature-controlled hotplate maintained at 36.4 °C, and thermal images were acquired from a fixed distance to ensure consistency across measurements. The preliminary evaluation of the TLC-based vein visualization patch was conducted on three healthy adult volunteers (two females and one male): a 32-year-old female with brown skin tone (BMI: 27.5 kg/m^2^), a 24-year-old female with fair skin tone (BMI: 22.0 kg/m^2^), and a 34-year-old male with brown skin tone (BMI: 24.6 kg/m^2^). The patch-on-skin images were captured using the built-in camera of a Google Pixel 7 smartphone. This feasibility study aimed to demonstrate superficial vein visualization under physiological skin temperature conditions and was not intended as a clinical validation.

## 3. Results and Discussion

### 3.1. Composition-Driven Thermochromism in TLCs

The five ternary TLC formulations (TLC1–TLC5) were designed by systematically varying the relative fractions of CN and CB while keeping COC constant at 32.5 wt.% (Table 2). The compositional window over which these three TLC components exhibit thermochromic response within the physiological temperature range was initially identified based on previously reported studies [40,41]. Building on this established range, the relative proportions of CN and CB were subsequently adjusted in a controlled manner to fine-tune the temperature-dependent optical behavior. This compositional design allows assessment of how small adjustments in the CN–CB-COC ratio influence pitch sensitivity and color-play behavior within the physiological temperature range. As CN content gradually increases from TLC1 to TLC5 (57.5 → 59.5 wt.%), CB content decreases in parallel (10 → 8 wt.%), enabling a controlled modulation of molecular packing and the temperature dependence of the cholesteric pitch.

**Figure 3:**
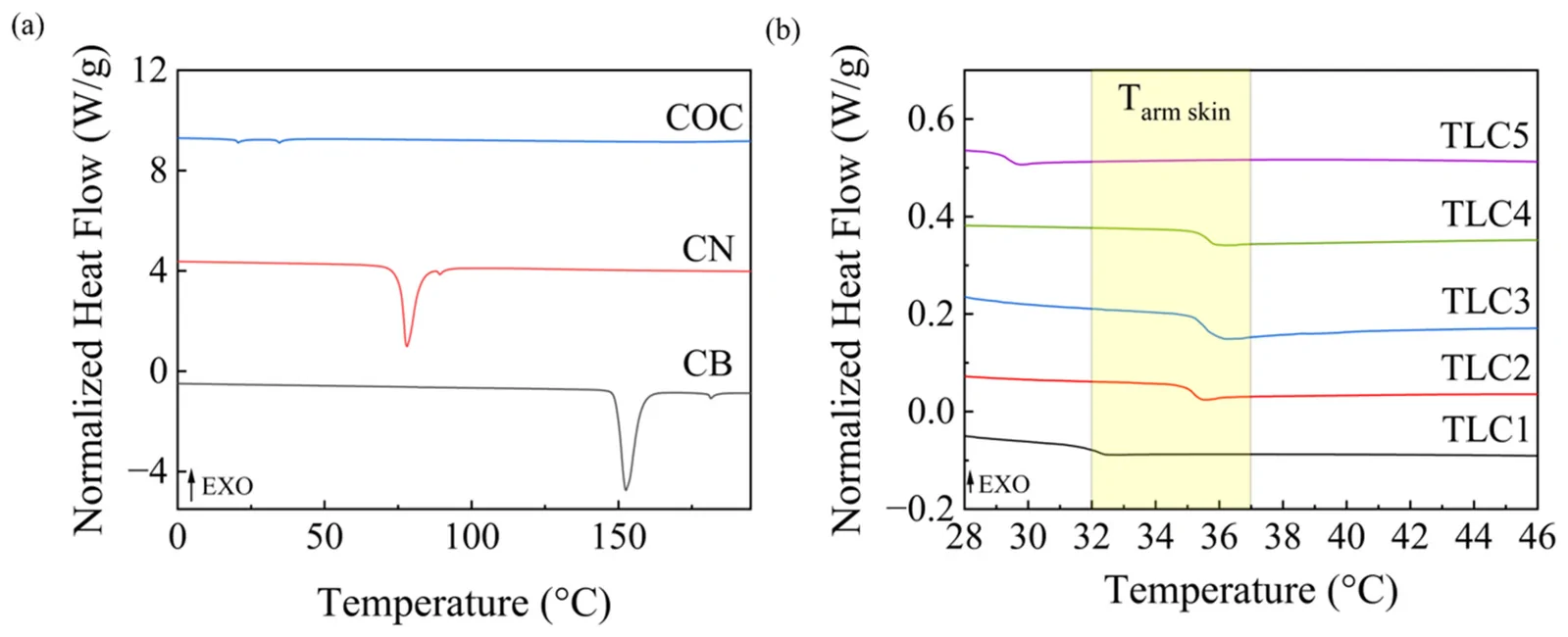
DSC curves of (**a**) each TLC component and (**b**) ternary mixture compositions at constant COC = 32.5% content. The normalized heat flow has been shifted for better illustration. Measurements were conducted at a heating rate of 20 °C min^−1^, and phase transformations were analyzed from the second heating cycle.

Figure 3 presents the DSC thermograms of the individual TLC components and their ternary mixtures. The pure components exhibit distinct thermal behaviors that reflect their molecular structures. CB shows a sharp, high-temperature melting peak at 152.6 °C, characteristic of its rigid aromatic backbone. At a higher temperature (~181.5 °C), CB also exhibits a phase change that corresponds to its clearing temperature, above which liquid crystalline order is lost, leaving an isotropic liquid [53]. CN displays a lower-temperature melting endotherm at 77.9 °C, consistent with its greater conformational flexibility. It also exhibits a second thermal transition at 89.3 °C, corresponding to its clearing temperature [54]. In contrast, COC exhibits a broad, low-intensity thermal response, indicating mesophase stabilization over an extended temperature range. Specifically, COC shows two endothermic transitions at approximately 20.6 °C and 34.6 °C, corresponding to the smectic–cholesteric and cholesteric–isotropic phase transitions, respectively [55]. A magnified view of these transitions is provided in Figure S3 of the Supplementary Information. When combined into ternary mixtures with a constant COC content of 32.5 wt.%, the sharp thermal transitions associated with CN and CB are suppressed. As a result, TLC1–TLC4 formulations exhibit smooth, broadened DSC profiles with mesophase transition temperatures falling within the physiological skin-temperature range (≈32–37 °C) [33,56,57]. The absence of distinct thermal events within this window confirms that the mixtures remain in a stable cholesteric phase under operating conditions. This behavior can be attributed to the structural compatibility between CN and COC, which both possess flexible aliphatic chains and similar steric characteristics [58]. Their molecular similarity promotes efficient packing and high miscibility within the cholesteric mesophase, thereby suppressing abrupt phase transitions. In contrast, CB contains a rigid benzene ring, introducing steric hindrance that locally disrupts mesophase packing [59]. At higher CB contents (e.g., TLC1), this rigidity contributes to enhanced thermal stability by restricting molecular mobility and stabilizing the cholesteric helical structure, as evidenced by a flatter DSC baseline and reduced thermal drift.

**Figure 4:**
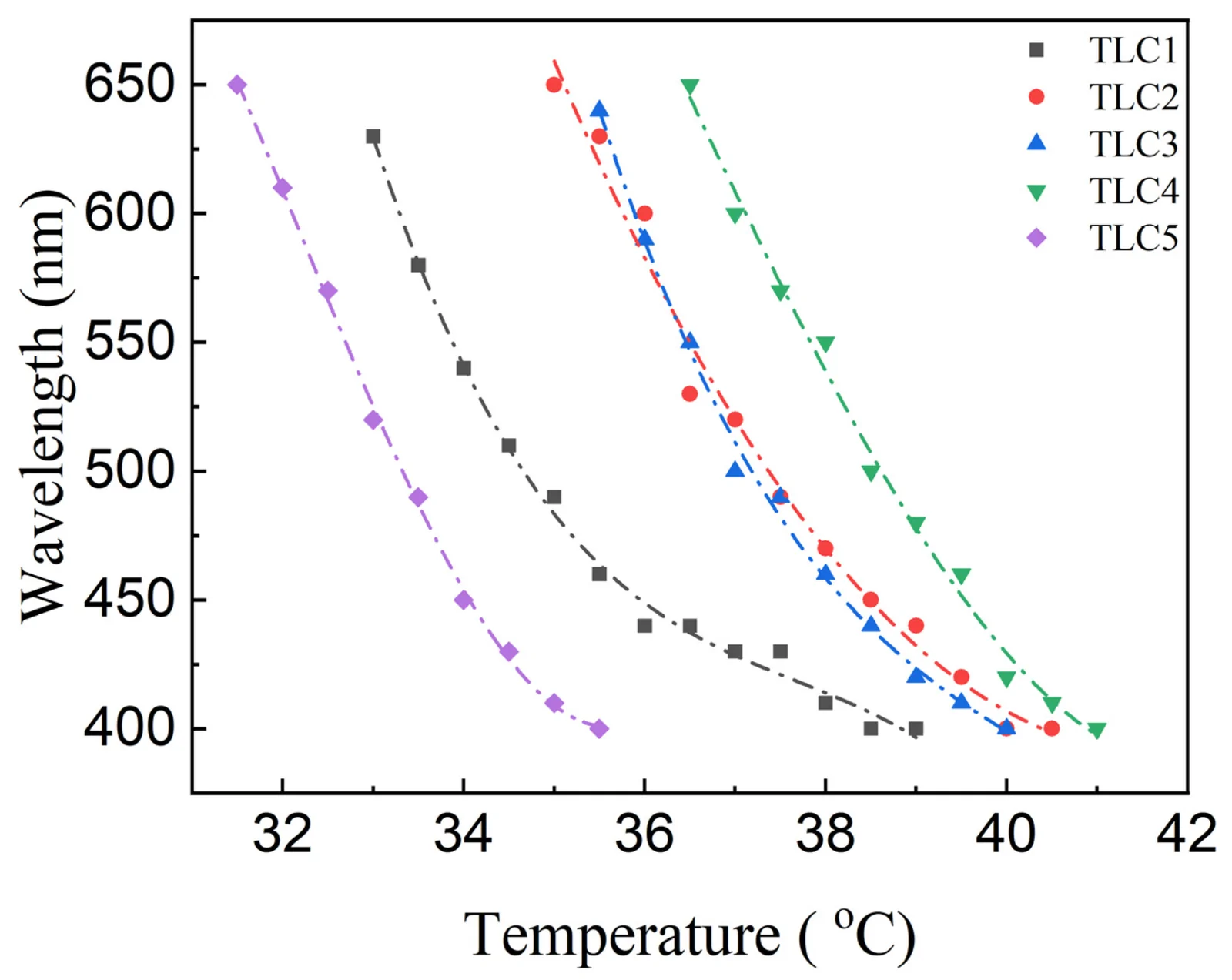
Maximum visible reflectance wavelengths of the TLC samples vs. temperature.

Figure 4 shows the reflected wavelength as a function of temperature for the five ternary TLC mixtures. All samples exhibit the expected blue-shift in reflection with increasing temperature, consistent with thermally induced pitch contraction in cholesteric liquid crystals. However, the rate of wavelength change varies systematically with composition. The blue-phase sensitivity, defined as the rate of change in the reflected peak wavelength with temperature in the blue spectral region (nm °C^−1^, Table 3), increases markedly from TLC1 to TLC5 (22.5 → 45 nm °C^−1^), reflecting the gradual increase in CN content and corresponding decrease in CB. Increasing the CN fraction while reducing the CB content alters intermolecular packing and the equilibrium cholesteric helical structure. Variations in steric interactions and molecular organization affect the helical pitch and its temperature dependence, thereby influencing the wavelength sensitivity of TLCs [35]. The change is CN/CB ratio is expected to influence molecular packing and the balance of chiral interactions within the mixture. These composition-dependent structural variations may increase pitch responsiveness at shorter wavelengths, resulting in the larger blue-phase slope observed for TLC5.

The high coefficients of determination (R^2^ = 0.90–0.98), which quantify how well the reflected peak wavelength varies linearly with temperature, confirm predictable temperature-dependent optical behavior across all mixtures. Interestingly, the red-to-green sensitivities, defined analogously as the slope of reflected peak wavelength versus temperature corresponding to the red-to-green color transition, are higher overall (75–86.7 nm °C^−1^), consistent with stronger pretransitional pitch expansion at longer wavelengths. TLC1 exhibits the highest red-to-green sensitivity (86.7 nm °C^−1^), whereas TLC5 shows the highest blue-phase sensitivity (45 nm °C^−1^). These observations suggest that compositional variations do not simply enhance or reduce the overall thermochromic sensitivity; rather, changes in CN/CB ratio shift the wavelength region over which the cholesteric helix exhibits the greatest temperature responsiveness. Overall, the results demonstrate that small compositional adjustments (≤1 wt.%) produce measurable changes in thermochromic sensitivity, enabling precise tuning of optical response within the physiologically relevant temperature range. The highest red-to-green sensitivity of TLC1 and its active temperature zone make it the most suitable candidate to visualize veins under physiological temperatures.

**Figure 5:**
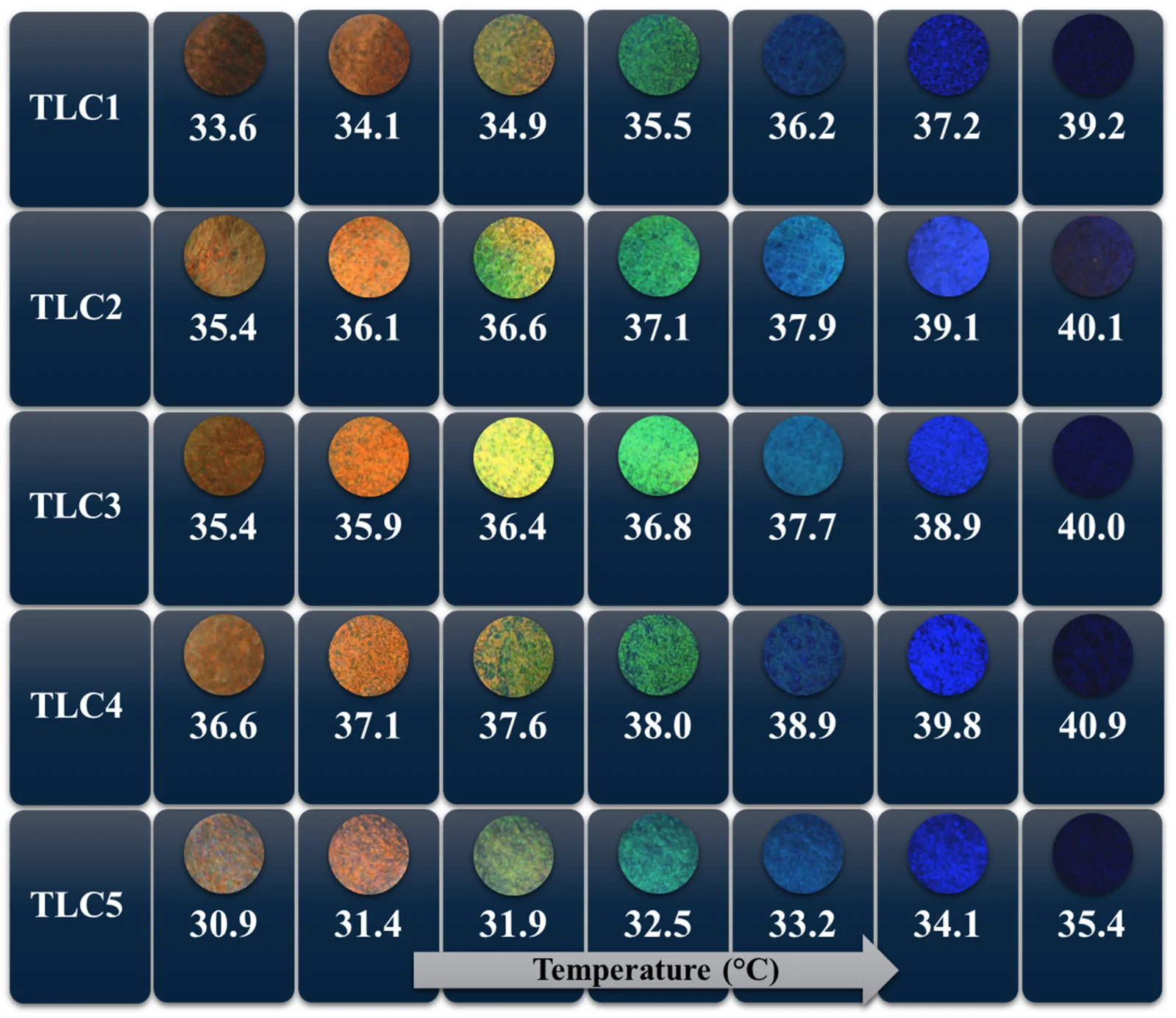
Color variations in the TLC ternary mixtures under OM within their mesophase temperature changes (red start, maximum red, green start, maximum green, blue start, maximum blue, blue end). Thermochromic temperature ranges of each TLC formulation are as follows: TLC1: 33.6–39.2 °C; TLC2: 35.4–40.1 °C; TLC3: 35.4–40.0 °C; TLC4: 36.6–40.9 °C; TLC5: 30.9–35.4 °C.

Figure 5 shows OM images capturing the thermochromic response of the five ternary TLC formulations across their mesophase temperature ranges. The observed color changes are consistent with the visible reflectance wavelength shifts measured as a function of temperature, confirming that the optical response arises from thermally induced contraction of the cholesteric helical pitch. All formulations maintain stable cholesteric ordering within their operational windows.

Clear compositional differences are observed among the formulations. Among our experimental conditions, TLC1 is the only composition that exhibits color changes spanning the physiological skin-temperature range of approximately 32 to 37 °C and presents sharp, well-defined color transitions within this interval. In contrast, TLC5 displays an earlier onset of color change and a dominant blue response at higher temperatures. The uniform textures and consistent color saturation observed in the OM images indicate homogeneous molecular alignment and support a reliable thermochromic response across all samples, with TLC1 uniquely suited for skin-relevant thermal sensing.

**Figure 6:**
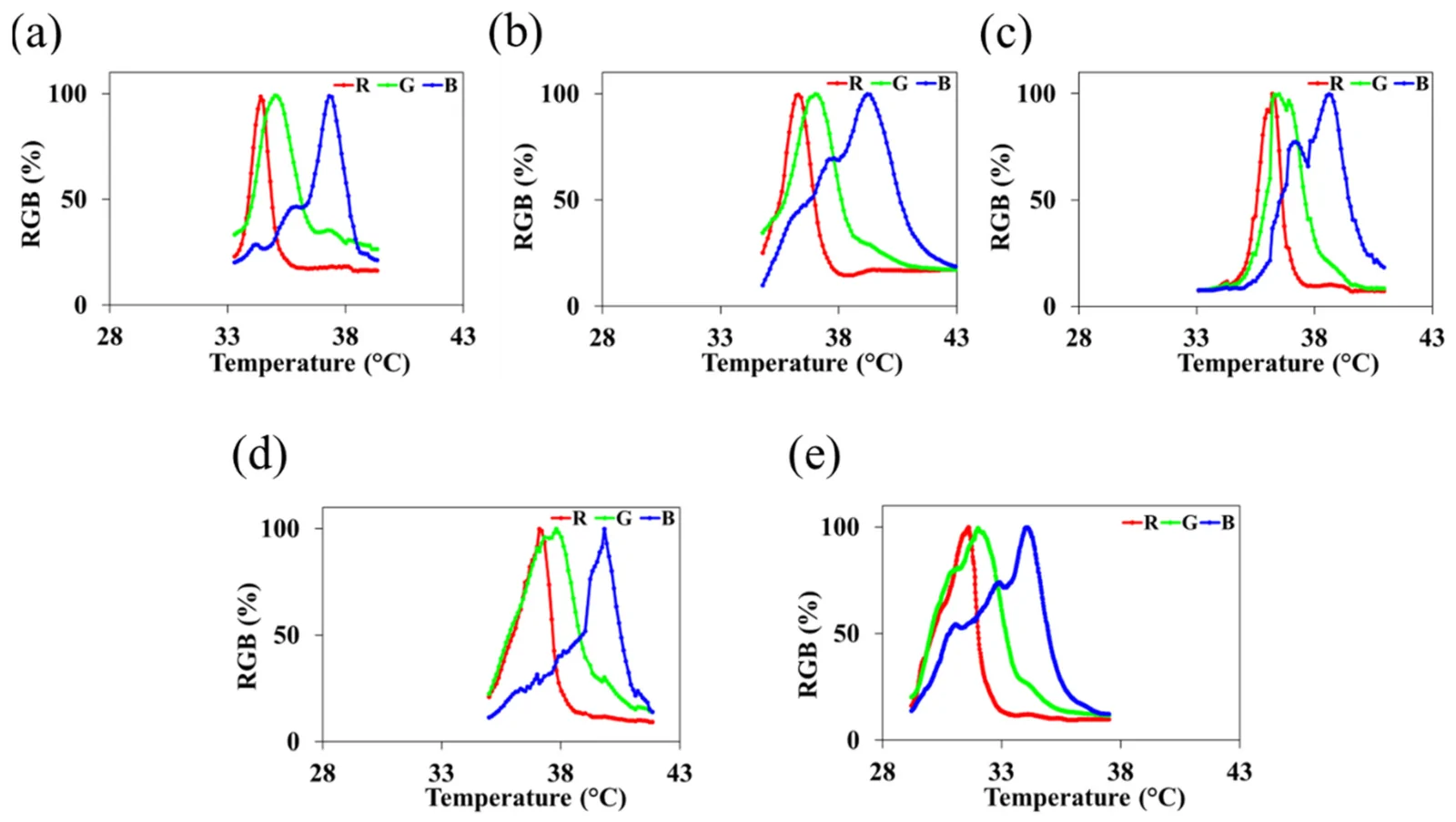
RGB diagrams of ternary TLC mixtures as a function of temperature, obtained by extracting red, green, and blue intensity values from optical microscopy color-change videos using MATLAB (version R2021a): (**a**) TLC1, (**b**) TLC2, (**c**) TLC3, (**d**) TLC4, and (**e**) TLC5.

Figure 6 shows the RGB intensity profiles of the five ternary TLC formulations as a function of temperature, providing a quantitative description of the thermochromic behavior observed in the OM images. The evolution of the RGB signals reflects the same temperature-driven contraction of the cholesteric helical pitch inferred from the OM color changes. At the onset of coloration, the red channel dominates, indicating reflection at longer visible wavelengths. With increasing temperature, the red intensity decreases while the green component reaches a maximum, corresponding to intermediate pitch values. Upon further heating, the blue intensity increases markedly, consistent with reflection at shorter wavelengths before the reflected color moves outside the visible range.

Although all mixtures exhibit the expected color transition from red through green to blue, their thermal response windows differ notably, reflecting the influence of composition on pitch sensitivity.

These differences demonstrate how tailored ratios of COC, CN, and CB govern thermal sensitivity within the physiological temperature range. A formulation exhibiting strong red and green responses near 33 to 35 °C, such as TLC1, is particularly effective for visualizing small temperature differences relevant to skin and underlying vein regions. In this composition, the overlap and rapid transition of the RGB channels within the physiological window provide enhanced contrast and high-temperature resolution, enabling accurate spatial discrimination of thermal features. By contrast, formulations with red and green peaks occurring outside the physiological range, such as TLC5, show reduced sensitivity in this critical interval and are therefore less suitable for threshold-based or contrast-driven skin-temperature sensing. Among the formulations, TLC1 uniquely aligns its red-to-green transition with the 32–37 °C physiological window, making it the most suitable candidate for vein visualization.

### 3.2. Breathability and Mechanical Compliance

While the thermochromic response determines the vein visualization capability of the TLC-embedded patch, its practical application as a wearable skin sensor also requires appropriate functional characteristics to ensure user comfort and mechanical adaptability. In particular, moisture transport through the patch is important for maintaining skin comfort, whereas mechanical compliance is essential for conformal contact with the skin during movement. Therefore, the water vapor transmission and mechanical properties of the TLC-embedded films were subsequently evaluated.

**Figure 7:**
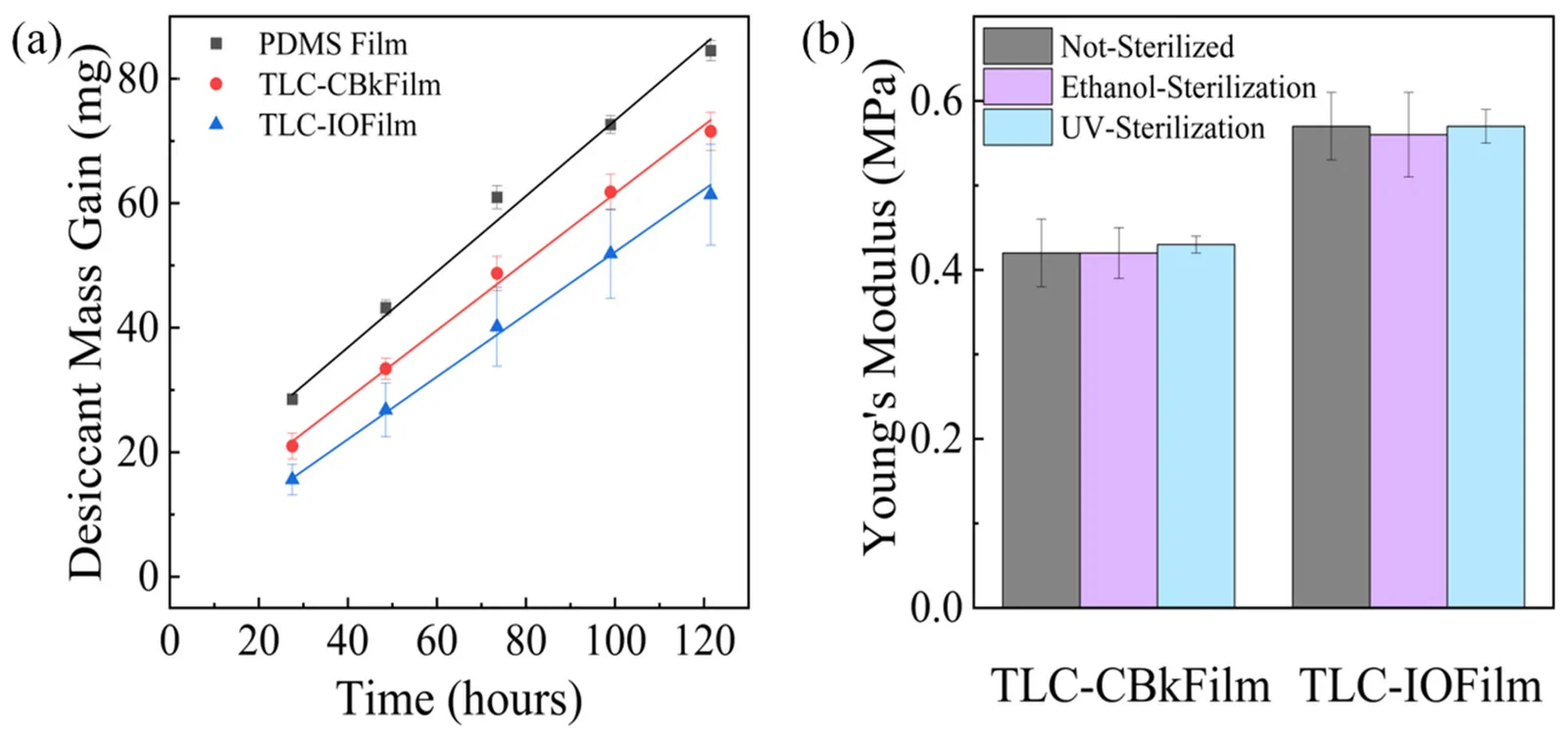
Diagrams of (**a**) water permeation with respect to time, and (**b**) elastic modulus of the TLC-embedded films containing the PDMS-based substrates with the black additives carbon black (TLC-CBkFilm) or iron oxide (TLC-IOFilm), subject to non-sterilized (control) and sterilized (either ethanol or UV) conditions.

Figure 7a compares the time-dependent desiccant mass gain for neat PDMS and TLC-embedded films incorporating carbon black (TLC-CBkFilm) and iron oxide (TLC-IOFilm). The control PDMS exhibits the highest mass uptake over time, consistent with the intrinsically high vapor permeability of cross-linked PDMS [60]. In comparison, both additive-containing films show a slight reduction in mass gain across the full 120 h period, indicating lower but still sustained water vapor transmission. The water vapor transmission rate (WVTR) was quantified from the slope of the linear regression of desiccant mass gain versus time, normalized by the exposed film area. As summarized in Table 4, neat PDMS exhibited a WVTR of 73.64 ± 3.00 g m^−2^ day^−1^, whereas the incorporation of carbon black and iron oxide reduced the WVTR to 66.55 ± 2.29 g m^−2^ day^−1^ and 60.75 ± 1.47 g m^−2^ day^−1^, respectively. These results indicate that the addition of CB and IO fillers decreases WVTR by approximately 9.6% and 17.5%, respectively, compared with neat PDMS. The reduced WVTR observed in the TLC-CBFilm and TLC-IOFilm can be attributed to the presence of dispersed particulate fillers, which introduce additional diffusion resistance by increasing the tortuosity of the vapor transport pathway without fully obstructing the polymer network. Importantly, the permeation profiles remain linear and progressive throughout the measurement period, indicating that the films maintain measurable water vapor transport characteristics. This moderate reduction in vapor transmission may help regulate moisture accumulation during skin contact while maintaining the structural integrity required for wearable sensing applications.

Since the developed TLC-embedded patch is intended for potential skin-contact applications, maintaining mechanical compliance after sterilization exposure is an important consideration. Sterilization procedures may influence the crosslinked PDMS network or filler–matrix interactions, potentially affecting the flexibility and conformability of the patch. Therefore, the Young’s modulus of the TLC-embedded films was evaluated before and after exposure to commonly used sterilization protocols (ethanol immersion and UV irradiation) to assess whether these treatments influence the mechanical properties of the materials [61]. Figure 7b shows the Young’s modulus of the TLC-embedded films before and after sterilization exposure. Both TLC-CBkFilm and TLC-IOFilm exhibit elastomeric moduli in the range of approximately 0.42–0.58 MPa, which is comparable to reported values for human forearm skin (0.42–0.75 MPa) [62]. Mechanical compliance within this range is critical for wearable thin-film sensors, as intimate skin adhesion and deformation compatibility require elastic moduli comparable to that of skin, typically below 1 MPa [63,64]. Importantly, neither ethanol nor UV exposure resulted in a statistically significant change in modulus, suggesting that the tested sterilization conditions had a limited effect on the mechanical properties of the crosslinked PDMS network and filler-containing films. The slightly higher modulus observed for TLC-IOFilm compared with TLC-CBkFilm is attributed to the increased stiffness of iron oxide particles and their interactions with the PDMS matrix [65]; however, the modulus remains within the range suitable for mechanically compliant wearable materials. Overall, the results indicate that the TLC-embedded films retain their low modulus and mechanical compliance following the evaluated sterilization treatments. These findings provide an initial assessment of the material response to sterilization exposure; however, further studies involving comprehensive sterilization validation and long-term reliability testing are required for biomedical translation.

### 3.3. Temperature Distribution and Vein Visualization

**Figure 8:**
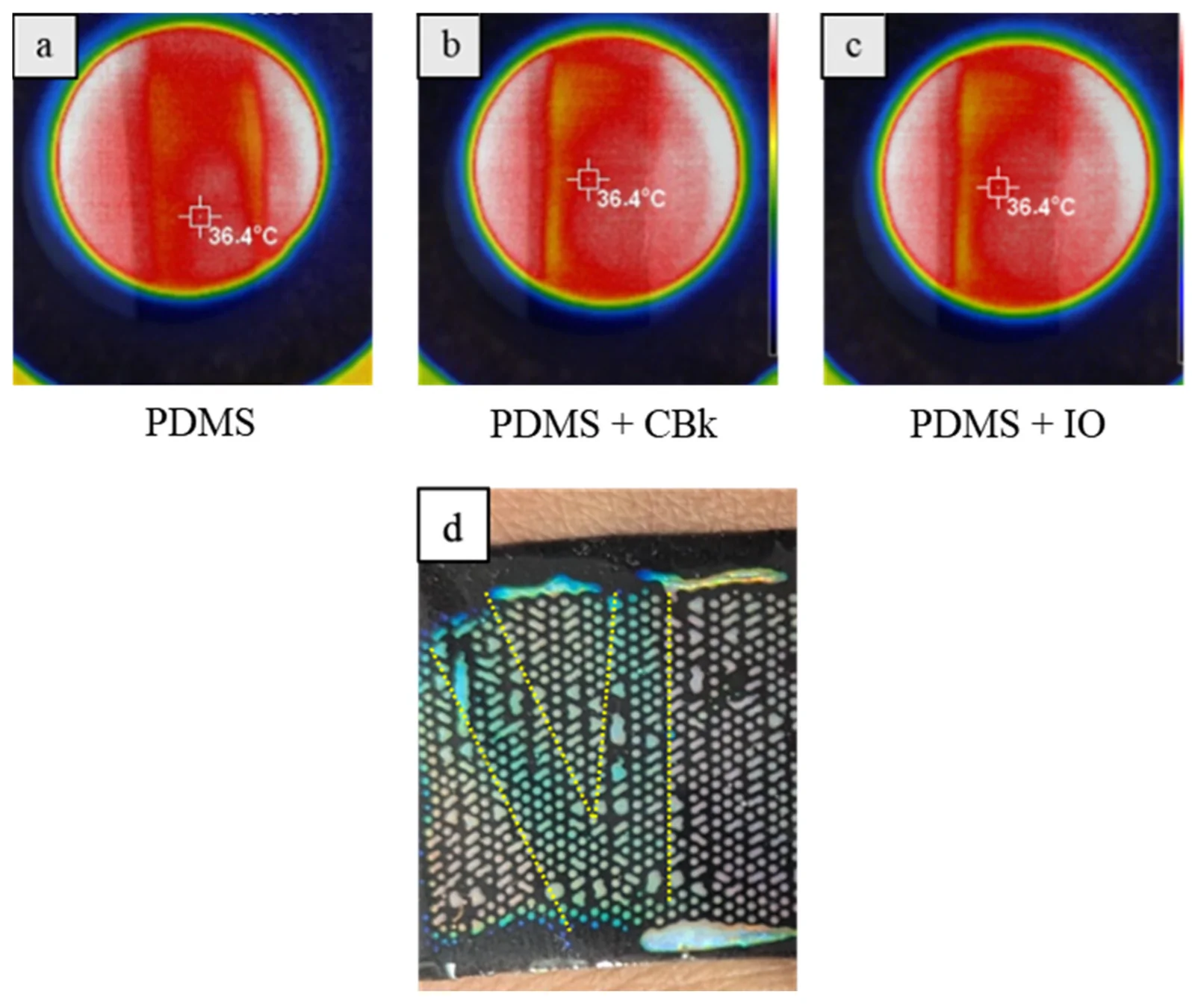
(**a**–**c**) Temperature distribution across different substrate layers (neat, CBk, and IO), demonstrating uniform thermal dispersion through infrared thermography. (**d**) Applying TLC-embedded films with CBk additive on forearm.

Figure 8a–c compares the infrared temperature distributions of PDMS substrates containing no additive and those incorporating CBk, and IO within the physiological temperature range (36.4 °C). All substrates exhibit spatially uniform temperature profiles with no detectable hot spots or gradients, demonstrating that the inclusion of optically absorptive and thermally conductive fillers does not perturb thermal homogeneity. This behavior directly reflects the effectiveness of the ultrasonication-based dispersion process.

High-energy pulsed ultrasonication generates localized cavitation and shear forces that disrupt agglomerates and promote homogeneous dispersion of CBk and IO within the PDMS matrix prior to cross-linking. This is particularly important for fillers such as CBk, which exhibit strong van der Waals-driven aggregation, and for IO particles that can additionally interact magnetically. The absence of localized thermal anomalies in Figure 8 suggests an effective dispersion of the fillers within the PDMS matrix. The observed thermal uniformity is consistent with the optical microscopy observations (Figure S4), where CBk and IO fillers showed relatively uniform distribution without significant macroscopic agglomeration. Although minor clustering was observed for IO, no corresponding localized thermal anomalies were detected, suggesting that the filler distribution remained sufficient to maintain consistent heat transfer across the substrate. This thermal homogeneity is important for TLC sensing because it minimizes substrate-induced variations and allows the observed color changes to more reliably reflect localized temperature differences arising from the skin–vein interface.

Figure 8d shows the application of TLC1-embedded PDMS film with CBk additive on the forearm. The image demonstrates the ability of the patch to reveal superficial veins, with the naturally occurring thermal contrasts along the skin clearly visualized through the color changes in the TLCs. Quantitative image analysis using ImageJ (version 1.54). revealed a signal-to-background ratio (SBR) of 1.36, calculated from the mean pixel intensities of the vein region and the surrounding tissue region, demonstrating the distinguishable contrast between superficial veins and adjacent skin.

It is important to note that this composition does not universally ensure vein visualization across all individuals. Human skin temperature is inherently variable and influenced by multiple physiological and environmental factors, including ambient conditions, local blood perfusion, metabolic activity, skin thickness, and hydration levels [66,67]. As a result, a TLC formulation that produces optimal contrast in one individual may not yield the same performance in another. Therefore, effective vein visualization using TLC-based films requires tuning of the thermochromic response range to align with the specific baseline skin temperature and thermal gradients of the target user. This highlights the need for customizable TLC compositions to ensure consistent performance across diverse users. Further investigations involving a broader range of physiological conditions, including variations in peripheral circulation, tissue thickness, and skin–vein thermal contrast, as well as cytocompatibility and skin irritation assessments, will be necessary to evaluate the clinical applicability and reliability of TLC-based vein visualization in diverse patient populations.

## 4. Conclusions

PDMS-based thermochromic skin patch was developed incorporating TLCs for visualization of superficial veins via natural thermal contrasts. Systematic tuning of ternary TLC mixtures enabled precise control of mesophase stability, color-play, and temperature sensitivity, with sub-percent compositional changes producing predictable thermochromic shifts. The optimized patches demonstrated reversible wavelength–temperature responses within the physiological temperature range. Patterned TLC microdomains on ultrasonicated PDMS yielded mechanically compliant, breathable films with uniform thermal transmission and stability under ethanol and UV exposure, while skin-matched elasticity ensured conformal contact and efficient thermal coupling. While this study focuses on qualitative vein visualization, future work will quantify spatial resolution and evaluate long-term on-skin performance under dynamic conditions. These results establish a scalable, compositionally tunable thermochromic elastomer platform for converting subtle physiological temperature variations into visible optical contrast. Beyond vein visualization for home hemodialysis, this system offers broad potential for wearable thermal mapping, skin-integrated diagnostics, and passive physiological sensing.

## Figures and Tables

**Table 1:** Current Technologies for AVF and Vein Visualization in Home Hemodialysis: Strengths and Limitations.

Technology	Working Principle	Advantages	Limitations for Home Hemodialysis AVF Visualization	Power/Hardware Requirement	Suitability for Wearable AVF Monitoring
Skin markers/tattoos [15,19,20,21]	Permanent or semi-permanent marking of vessel location after clinical mapping	Simple, low cost, no electronics required	Fixed location; does not account for changes in AVF position, vessel enlargement, or access site variation; requires repeated clinical marking	None	Limited
Ultrasound imaging [22,23,24,25]	Acoustic imaging of vessel structure and depth	Provides detailed information on vessel diameter, depth, and flow characteristics	Requires trained personnel; bulky equipment; relatively high cost; not practical for routine self-use at home	Ultrasound probe and imaging system	Moderate (mainly clinical use)
Near-infrared (NIR) vein visualization [24,26,27]	Detects optical absorption contrast of hemoglobin to visualize superficial vessels	Non-invasive, rapid visualization of superficial veins	Requires camera, NIR illumination, and image processing; typically designed for peripheral veins; limited portability and continuous monitoring capability; performance may decrease for deeper AVF vessels	Battery/electronic imaging components	Moderate (mainly clinical use)
Infrared thermal imaging [28,29,30]	Detects infrared radiation emitted from the skin to generate temperature maps	Non-contact imaging; provides high-resolution surface temperature distribution; can identify thermal patterns associated with superficial vasculature	Requires specialized thermal camera, calibration, and image processing. Vein-related temperature patterns are displayed on the camera screen rather than directly on the skin, requiring the user to translate the displayed image to the cannulation site. This limits hands-free guidance during self-cannulation and continuous wearable use	Infrared thermal camera and imaging software	Low–Moderate
TLC-based thermochromic skin patch (this work)	Temperature-responsive cholesteric liquid crystal reflection induced by local thermal variations associated with vascular structures	Passive, lightweight, flexible, wearable; direct visual readout; no external power, camera, or electronic processing required; suitable for repeated home use	Provides indirect visualization based on thermal contrast; requires optimization for individual skin conditions and vascular depth	No external power or electronics	High potential for home-based AVF monitoring

**Table 2:** Ternary mixture compositions of the TLC materials: Cholesteryl Nonanoate (CN), Cholesteryl Benzoate (CB), and Cholesteryl Oleyl Carbonate (COC).

Sample	CN (wt.%)	CB (wt.%)	COC (wt.%)
TLC1	57.5	10.0	32.5
TLC2	58.0	9.5	32.5
TLC3	58.5	9.0	32.5
TLC4	59.0	8.5	32.5
TLC5	59.5	8.0	32.5

**Table 3:** Sensitivity of the TLC samples in their blue phase and red-to-green color change, with respect to temperature, calculated from reflected wavelength vs. temperature.

Sample	Blue Phase Sensitivity (nm °C^−1^)	R^2^	Red-to-Green Sensitivity (nm °C^−1^)	R^2^
TLC1	22.5	0.90	86.7	0.99
TLC2	30	0.98	83.33	0.96
TLC3	36	0.95	75	0.97
TLC4	40	0.96	75	0.98
TLC5	45	0.94	80	0.99

**Table 4:** WVTR and thickness-normalized WVTR of PDMS-based films.

Sample	WVTR (g m^−2^ Day^−1^)	Thickness (mm)	Thickness-Normalized WVTR (WVTR × Thickness) (g·mm·m^−2^·Day^−1^)
PDMS Film	73.64 ± 3.00	0.22512	16.57± 0.68
TLC-CBk Film	66.55 ± 2.29	0.22926	15.26 ± 0.53
TLC-IO Film	60.75 ± 1.47	0.22926	13.93± 0.34

## Data Availability

The data supporting the findings of this study are contained within the manuscript.

## Supplementary Materials

The following supporting information can be downloaded at: https://www.mdpi.com/article/10.3390/polym18161934/s1. Figure S1. (a) Optical photograph and (b) optical microscopy image of the laser-cut stencil used in skinpatch fabrication, Figure S2. Enlarged DSC thermogram of COC highlighting the smectic–cholesteric transition at ~20.6 °C and the cholesteric–isotropic transition at ~34.6 °C, Figure S3. Schematic illustration of the three-layer architecture consisting of the black substrate, thermochromic liquid crystal layer, and transparent protective layer, and the final assembled wearable thermochromic skin patch, Table S1. Mesophase Transformation Temperatures of TLC1–TLC5, Figure S4. Optical microscopy images of filler-dispersed PDMS substrates. (a) CBk-PDMS and (b) IO-PDMS after ultrasonication-assisted dispersion.
