# Supplementary material for: TLC-Embedded PDMS-Based Wearable Skin Patch for Vein Visualization Applications
Source: Polymers (Basel). 2026 Aug 7;18(16):1934. doi: 10.3390/polym18161934 (PMC13516250; doi:10.3390/polym18161934)
Supplement: Supplementary file 1 [file polymers-18-01934-s001.zip › polymers-4329890-supplementary.pdf]

Supplementary Information

TLC-embedded PDMS-based wearable skin patch for vein visualization applications

Mayesha Binte Mahmud[1], Yalda Chehrehsaz[1], Rafaela Aguiar[1], Anthony V. Tuccitto[1] , Lei Zhang[1], Vivian Luu[1], Isabella Terefenko[1], Katherine Jia[1], Xia Wei Shen[1], Christopher T. Chan[2], Patrick C. Lee[1*]

[1]Department of Mechanical and Industrial Engineering, University of Toronto, 5 King's College Road, Toronto M5S 3G8, Canada
[2]University Health Network, Department of Medicine, Division of Nephrology, 200 Elizabeth Street, 8N room 846, Toronto, ON M5G 2C4, Canada
* Corresponding Author: Patrick.c.lee@utoronto.ca

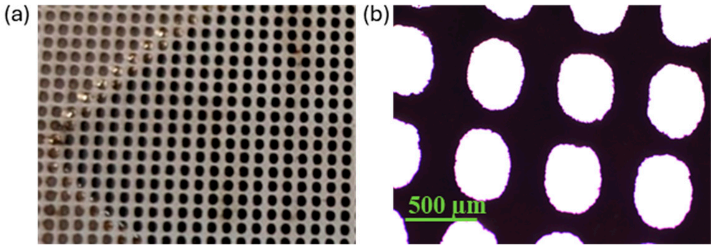

Figure S1. (a) Optical photograph and (b) optical microscopy image of the laser-cut stencil used in skinpatch fabrication.

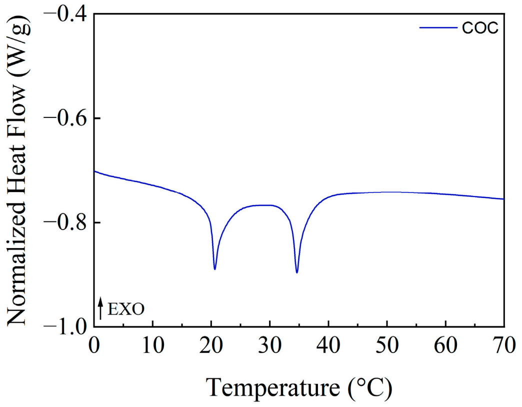

Figure S2. Enlarged DSC thermogram of COC highlighting the smectic–cholesteric transition at ~20.6 °C and the cholesteric–isotropic transition at ~34.6 °C.

Table S1. Mesophase Transformation Temperatures of TLC1–TLC5

Sample	Mesophase Transformation Temperature (°C)
TLC1	32.12 ± 0.11
TLC2	35.24 ± 0.29
TLC3	35.58 ± 0.19
TLC4	35.63 ± 0.13
TLC5	29.41 ± 0.24

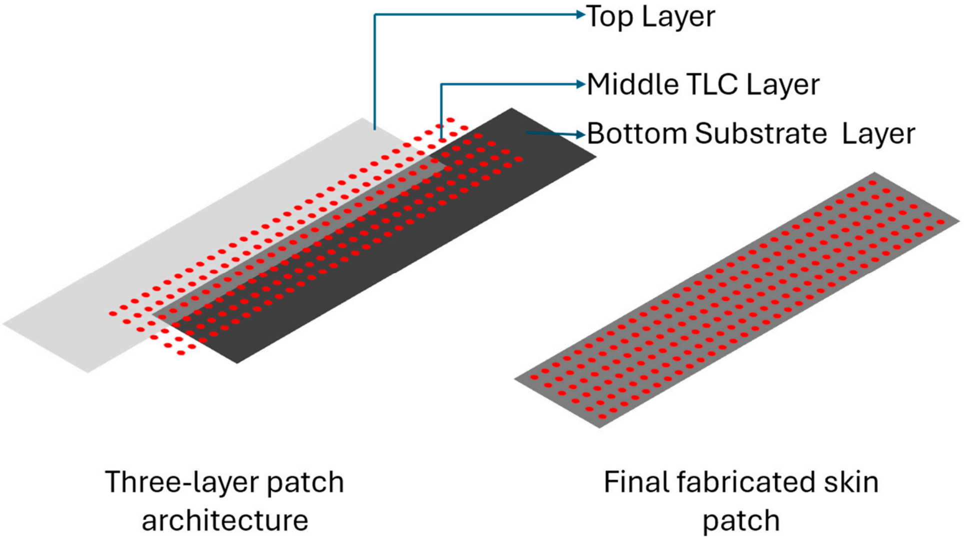

Figure S3. Schematic illustration of the three-layer architecture consisting of the black substrate, thermochromic liquid crystal layer, and transparent protective layer, and the final assembled wearable thermochromic skin patch.

TLC patches response time was evaluated using polarized optical microscopy (POM). The patches were placed on a temperature-controlled heating stage, and their color transition response was monitored during rapid heating at a rate of 60 °C min^{-1}. The time required for the TLC dots to reach the target color corresponding to the new temperature was recorded. The CBk-substrate and IO-substrate patches exhibited comparable response times, requiring approximately 6 s for the color transition when the temperature was increased from 35 to 36 °C.

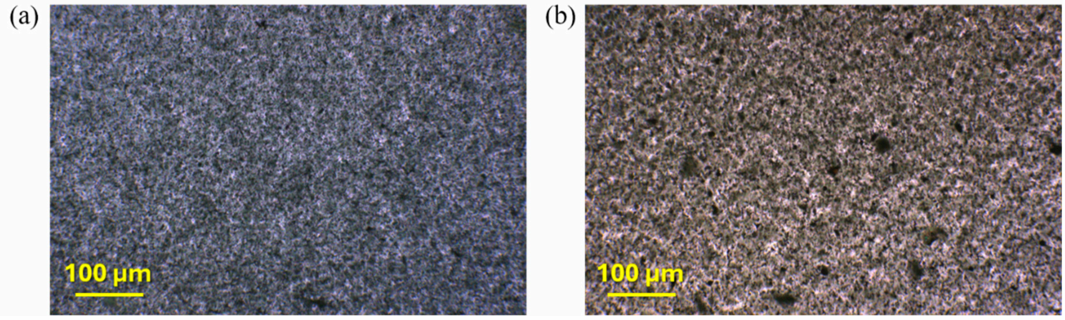

Figure S4. Optical microscopy images of filler-dispersed PDMS substrates. (a) CBk-PDMS and (b) IO-PDMS after ultrasonication-assisted dispersion.
